# Severity and Determinants of Anemia in TB/HIV Coinfected Adults at Mekelle, Ethiopia: Hospital Based Retrospective Study

**DOI:** 10.1155/2023/5555030

**Published:** 2023-05-17

**Authors:** Kebede Embaye Gezae, Kiflom Hagos, Assefa Ayalew Gebreslassie

**Affiliations:** ^1^Department of Biostatistics, School of Public Health, College of Health Sciences, Mekelle University, Mekelle, Tigray, Ethiopia; ^2^Department of Medical Microbiology and Immunology, Biomedical Division, School of Medicine, College of Health Sciences, Mekelle University, Mekelle, Tigray, Ethiopia; ^3^Department of Reproductive Health, School of Public Health, College of Health Sciences, Mekelle University, Mekelle, Tigray, Ethiopia

## Abstract

**Background:**

Anemia has up to 87% prevalence in high tuberculosis (TB) and human immunodeficiency virus (HIV) burden settings of the sub-Saharan Africa (SSA) including Ethiopia. It increases lost to follow-up (LTFU) rate, reduces quality of life, and shortens the survival of TB/HIV coinfected patients. However, there is limited information on severity level and determinants of anemia in TB/HIV coinfected adults in the study setting in particular. Therefore, this study is aimed to assess severity level and determinants of TB/HIV-associated anemia.

**Methods:**

A hospital based retrospective study was conducted among 305 TB/HIV coinfected adults who enrolled for antiretroviral therapy (ART) from January, 2009 to December, 2016 in two public hospital of Mekelle, Ethiopia, by reviewing ART register. A multiple logit model was fitted to identify the baseline determinants of anemia using 95% confidence level or 5% level of significance for adjusted odds ratio (AOR).

**Results:**

In the current study, the cumulative baseline prevalence of anemia was 59.0% (95% CI: 53.3%–64.6%). Considering severity level, the prevalence was 6.2%, 28.2%, and 24.6% for severe, moderate, and mild anemia, respectively. Being female (AOR = 0.380; 95% CI: 0.226–0.640), body mass index (AOR = 0.913; 95% CI: 0.836–0.998) reduces the odds of developing anemia whereas baseline ambulatory functional status (AOR = 2.139; 95% CI: 1.189–3.846), bedridden functional status (AOR = 2.208; 95% CI: 1.002–4.863), HIV clinical stage III (AOR = 2.565; 95% CI: 1.030–6.384), and HIV clinical stage IV (AOR = 2.590; 95% CI: 1.006–6.669) increased the odds of developing anemia for TB/HIV coinfected adults.

**Conclusions:**

In the current study, significant TB/HIV-associated severe anemia was assessed which accounted for nearly one-ninth of all anemia cases while nearly half were moderate anemia. Therefore, curious attention has to be given for the management of TB/HIV-associated severe anemia in particular and anemia in general to reducing anemia associated bad outcomes most importantly death.

## 1. Introduction

According to the World Health Organization (WHO), anemia is defined as hemoglobin level's of <12.0 g/dl in women and <13.0 g/dl in men though other factors such as race, physiological status, and pregnancy status have to be taken into consideration [1]. Anemia is a global public health problem that affects an estimated one-fourth of the world's population, with an inconsistent discrimination of cases occurring in sub-Saharan Africa (SSA) [2, 3]. The burden of anemia increases the morbidity and mortality, decreases quality of life, and negatively affects productivity of women particularly [4].

Though etiology of anemia is multifactorial, evidences have been showed that HIV and tuberculosis (TB) are both strongly significantly associated with anemia. HIV and its viral proteins as well as immune suppressions during the natural course of HIV infection were found to be responsible for bone marrow suppression which most likely leads to HIV-associated anemia [5, 6]. Furthermore, tuberculosis (TB) plays a significant role in causing HIV-related anemia in SSA region including Ethiopia [7–9].

Anemia is frequently manifested among HIV patients of Antiretroviral Therapy (ART)-naïve in SSA, with a prevalence that ranged from 45% to 87% [10–13]. Anemia is one of the leading cause and frequent clinical complications of both HIV and TB diseases and it is associated with substantial morbidity and mortality in TB/HIV coinfected patients [14–16]. It is also related with markedly increased lost to follow-up (LTFU) rate of up to 23% in anemic TB/HIV coinfected adults as compared to nearly 10% among nonanemic adults at baseline in a study conducted in the same setting [17]. Reversely, anemia may serve as a useful biomarker for the HIV-associated TB diagnosis and clinical management of such patients [18]. On the other hand, anemia had reported to be strongly and consistently associated with HIV disease progression, suggesting the need for routine screening and treatment even in patients on highly active antiretroviral therapy (HAART) [19–21].

HIV-infected people with low CD4+ cell count, high erythrocyte sedimentation rate, and vitamin D deficiency was identified the main predictors of anemia [22, 23]. Literature has revealed that the risk of death was reached up to 70% or greater in HIV-infected anemic patients compared with nonanemic counterpart which significantly affects the quality of life [24].

To the level of our understanding, only few studies were conducted on HIV-associated anemia in Ethiopia, but none were in TB/HIV coinfected patients. Consequently, these studies reported the prevalence of anemia that varied from 23.0% to nearly 43.0% with the highest contribution being not on ART. Rural place of residence, WHO clinical stage III/IV, presence of TB coinfection, and low baseline CD4 cell count were reported as positive predictors of HIV-associated anemia in one and/or more of these studies [25–28]. However, none of the studies were assessed the severity level and baseline determinants of TB/HIV-associated anemia. Thus, this study is aimed to generate evidence on TB/HIV-associated anemia and its determinants in the current study setting.

## 2. Materials and Methods

### 2.1. Study Setting, Design, and Population

A baseline hospital based cross-sectional study was conducted among TB/HIV coinfected adults who started ART from September, 2009–December, 2016 at two governmental hospitals namely–Mekelle hospital and Ayder comprehensive specialized hospital (ACSH)–Mekelle, Ethiopia. Mekelle is the capital city of the national regional state of Tigray which is 783 kilometers far from Addis Ababa, the capital of Ethiopia. ART service has been begun since 2004 in Mekelle Hospital and since 2009 in ACSH. To date, the ART clinics are active to provide ART services, and above tens of thousands of HIV patients utilized the service collectively in the two hospitals. Data were extracted from March 1 to April 31, 2017 by reviewing ART registers. All active TB and HIV coinfected adults who fulfilled the eligibility criteria were included in the current study. TB/HIV coinfected adults who had no baseline measurement of hemoglobin were excluded from the current study analysis.

### 2.2. Sample Size Determination and Sampling Proportion

We used the single population proportion formula to estimate the minimum sample size considering the following key assumptions: 95% confidence level, 5% acceptable level of precision (margin of error), and overall prevalence of anemia (23%) among HIV patients irrespective of TB status conducted in Debre-Tabor, Ethiopia [28].

(1) $$\begin{array}{c} n\left(minimum\;sample\;size\;estimated\right)=\frac{{\left(z_{\left(\alpha /2\right)}\right)}^{2}pq}{d^{2}}\\ =\frac{1.96^{2}*0.23*0.77}{0.05^{2}}\approx 273\text{.} \end{array}$$

Eventually, 305 TB/HIV coinfected patient registers were reviewed in the current study using all consecutive sampling approach.

### 2.3. Study Variables, Tool, Data Quality, and Data Collection Procedure

A well-organized data abstraction sheet (checklist) was prepared and then modified considering the most important baseline socio-demographic and clinical characteristics of the target participants. After training on how to review data was given to data collectors, the following data elements were collected from the ART registers using a well standardized checklist:

1. Baseline socio-demographic characteristics of TB/HIV coinfected adults (sex, age, educational status, marital status, religion, and place of residence).
2. Baseline clinical and laboratory characteristics of the participants, i.e., functional status, WHO clinical stage, Body Mass Index (BMI) in kg/m^2^, serum alanine transferase (ALT) level, serum aspartate transferase (AST) level, and CD_4_+ cell count (in cells/mm^3^).
3. Hemoglobin concentration (g/dl) of TB/HIV coinfected adults. In the current study, hemoglobin was considered as the proxy indicator of anemia to classify the study subjects as nonanemic (hemoglobin ≥12 g/dl for women and ≥13 g/dl for men) or anemic (hemoglobin <12.0 g/dl for women versus <13.0 g/dl for men). Anemia is further classified in adult men as mild anemia (11.0–12.9 g/dl), moderate anemia (8.0–10.9 g/dl), and severe anemia (<8.0 g/dl). On the other hand, the classification of anemia is slightly different in adult women as mild anemia (11.0–11.9 g/dl), moderate anemia (8.0–10.9 g/dl), and severe anemia (<8.0 g/dl) [1]. Thus, anemia status is the outcome variable labeled as 1 if anemic (mild, moderate, or severe) and 0 otherwise (i.e., nonanemic).

The variable place of residence is operationalized as urban (i.e., for patients from Mekelle) or rural (i.e., for patients living outside Mekelle). Thus, some participants who were from urban area might be classified as rural considering the operational definition.

### 2.4. Statistical Methods of Data Analysis

First, data were entered, exported, and then cleaned in STATA version 14.2 statistical tool. The participants were described using frequency and percent. Serum ALT and AST levels, age, and BMI were described using median and interquartile range (IQR). The adequacy of cell count was also checked using cross-tabulation prior to run the binomial model. The severity of anemia was presented using bar graph. Model fitness and diagnostics (i.e., collinearity) were checked prior to declare the best fitted model. The logit model was fitted as a parsimonious model explaining the anemia data very well so that determinants of anemia were identified according to the final logit model fitted in the current study. Finally, the strength of association of each statistically significant predictor was measured and interpreted based on the adjusted odds ratio (AOR) and its corresponding 95% confidence interval (CI). For the categorical predictors, the AOR was interpreted in comparison to the reference category for the odds of developing anemia. On the other hand, for the continuous predictor particularly baseline BMI, the AOR was interpreted as a unit increase in the continuous predictor BMI decreases the odds of developing anemia by (1 − AOR) *∗* 100%.

### 2.5. Ethics Approval and Consent to Participate

Ethics approval was issued by the Institutional Review Board (IRB) of Mekelle University-College of Health Sciences, and the consent to participate was fully waived by the IRB since secondary data were extracted from HIV registers. However, data were kept confidentially, and no personal or identifiable information was used in the present study.

## 3. Results

### 3.1. TB/HIV-Associated Anemia Prevalence and Severity Level

**Figure 1:**
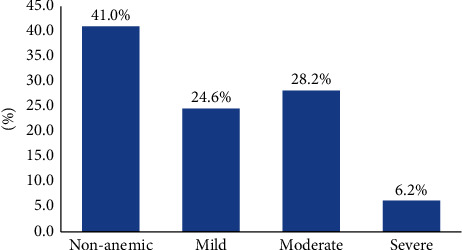
Severity of TB/HIV-associated anemia of patients who initiated ART in two public hospitals of Mekelle, Ethiopia, 2009–2016 (*n* = 305).

Based on the baseline hemoglobin measurement (g/dl) as a proxy indicator of anemia, anemia was reported in 180 (59.0%; 95% CI: 53.3%–64.6%) of TB/HIV coinfected adults. With regard to severity, moderate, and severe anemia were reported in 86 (28.2%; 95% CI: 23.2%–33.6%), and 19 (6.2%; 95% CI: 3.8%–9.6%) of TB/HIV coinfected adults, respectively. Nearly half (47.8%) of the anemia cases were moderate compared to 41.7% of mild and 10.6% of severe types (Figure 1).

### 3.2. The Severity of TB/HIV-Associated Anemia by Socio-Demographic Characteristics

In the current study, the overall prevalence of anemia in TB/HIV coinfected males was 68.2% as compared to 50.2% for females. However, the prevalence of severe form of anemia was significantly higher in females (7.6%) than in males (4.7%). The overall prevalence of anemia ranges from 52.1% (i.e., 8.4% severe anemia) in TB/HIV coinfected adults with secondary education to 66.7% (i.e., 5.6% severe) among adults with primary level of education. However, 75% of Muslim TB/HIV coinfected adults reported to have anemia with equal share of the three anemia levels–25% each. Though there was insignificant difference in the prevalence of anemia by place of residence, severe anemia had exceeded by 3% in patients from rural areas (Table 1).

### 3.3. The Severity of TB/HIV-Associated Anemia by Baseline Clinical Characteristics

The prevalence of anemia was linearly increased from 46.1% in adults with working functional status to 72.2% in adults with bedridden functional status. Likewise, the severe form of anemia was similarly increased from 2.8% in TB/HIV coinfected adults with working functional status to 14.8% in patients with bedridden functional status. The anemia prevalence was elevated as the baseline WHO clinical stage of HIV advanced from stage I or II (28.1%) to stage IV (67.6%) where the severe type of anemia was increased from 0% in adults with baseline stage I or II to 9.2% in stage IV. The prevalence of anemia was almost twice in patients with baseline CD4+ cell count ≤200 cells/mm^3^ as compared to patients with CD4+ cell count >200 cells/mm^3^ (64.0% versus 36.4%) in which 6.8% of patients with low CD4+ cell count tend to develop severe anemia. The median baseline BMI was 17.4 kg/m^2^ while the median serum ALT and AST levels were 34.5 and 40 IU/l, respectively (Table 2).

### 3.4. Determinants of TB/HIV-Associated Anemia

According to the final multiple logit model fitted, the baseline determinants of anemia were as follows: sex (female), functional status (ambulatory and bedridden), WHO clinical stage (III and IV), and BMI (kg/m^2^). Keeping other variables constant, female TB/HIV coinfected adults were protected by at least 35.6% and utmost 77.4% (62.0% on average) (AOR = 0.380; 95% CI: 0.226–0.640) from developing anemia than males. Reversely, the odds of developing anima among TB/HIV coinfected adults with baseline ambulatory functional status, bedridden functional status, WHO clinical stage III and IV were increased by 114% (AOR = 2.139; 95% CI: 1.189–3.846), 121% (AOR = 2.208; 95% CI: 1.002–4.863), 157% (AOR = 2.565; 95% CI: 1.030–6.384), and 159% (AOR = 2.590; 95% CI: 1.006–6.669), respectively, as compared to their respective reference categories. However, a unit increase in BMI (1 kg/m^2^) reduces the odds of developing anemia by almost 9% on average (AOR = 0.913; 95% CI: 0.836–0.998) in TB/HIV coinfected adults (Table 3).

## 4. Discussion

The current study was conducted to investigate the severity level and determinants of anemia in TB/HIV coinfected adults. Thus, it has been revealed that 59.0% (95% CI: 53.3%–64.6%) of adults coinfected with TB and HIV were anemic, and 6.2% had severe anemia at ART enrollment. Moreover, being female, baseline ambulatory functional status, baseline bedridden functional status, baseline WHO clinical stage III, baseline WHO clinical stage IV, and baseline BMI were identified as statistically significant determinants of baseline anemia in the current study.

In this study, the overall prevalence of anemia at baseline was found to be 59.0% (24.6% mild, 28.2% moderate, and 6.2% severe anemia) which almost exactly in line to findings obtained across Europe though the target groups were HIV patients of HAART naïve [29]. The current prevalence was higher than other studies that ranged from 18.9% in Uganda to 49.6% in Indonesia [25–28, 30–34]. Reversely, the baseline prevalence was lower than other studies conducted among ART naïve HIV or TB/HIV coinfected patients in Ghana, Nigeria, and Gambia that have been reported 63.0%, 69.2%, and 67.0%, respectively [11, 35, 36]. The reasons for the high prevalence of anemia at baseline in the current study are most probably related to the contribution of tuberculosis in speeding up the occurrence of HIV-associated anemia [7–9]. On the other hand, the low prevalence when compared to the other three studies could be the difference in operationalizing or defining anemia using hemoglobin level as proxy indicator and difference in ethnicity, geographic location, stage of the HIV infection, presence of other opportunistic infection, and feeding style of the population that might contributing a lot for the inconsistency of anemia prevalence.

In the current study, female sex is negatively statistically associated with the anemia prevalence in adult TB/HIV coinfected patients prior to ART initiation. The finding was in agreement with other studies conducted in Benin city (Nigeria) and Zewditu Memorial Hospital (Ethiopia) which have been reported all the low prevalence of anemia in females as compared to male HIV patients of ART naïve or ART experienced [11, 25]. However, it is inconsistent with other similar studies that have been reported the direct effect of female sex to anemia [26, 32, 33, 35]. The main reason for the inconsistent findings as compared to the current study finding might be the difference in definitions of anemia that used different cutoff values of hemoglobin for males and females across the different studies. Of course, logically the prevalence of anemia was expected to be higher on adult HIV and/or TB coinfected women because of the contributions of blood loss during menstruation, pregnancy, and delivery that aggravate the prevalence of anemia [22, 23]. Despite this fact, the prevalence of anemia was lower among adult TB/HIV coinfected women than men mainly due to (1) the cutoff point for anemia classification used for both sexes was not uniform (i.e., a woman with hemoglobin level of 12 g/dl and above is considered as normal whereas a man with hemoglobin level of 13 g/dl and above was considered as normal) and (2) there were other important but uninvestigated variables that might be confounded with sex such as feeding style and iron-folic acid supplementation that prevent the occurrence of anemia. Furthermore, the treatment seeking behavior to anemia and good feeding practices might be well exercised by females than males that most likely enriched during antenatal care (ANC) and postnatal care (PNC) follow-ups.

BMI (kg/m^2^) is also significantly negatively predicting the odds of developing anemia among TB/HIV coinfected adults. A unit increase in baseline BMI tends to protect the odds of developing anemia by almost 9% in the current study. This finding was consistent with other studies conducted in South Africa, Uganda, and Rwanda which all have supported the inverse relationship between baseline BMI and anemia [30, 33, 37]. However, the socio-demographic variable sex was fixed in the Rwandan study where the participants were only adult women. It is obvious that nutritional deficiencies including iron, folic acid, and vitamin B_12_ play a major role in causing nutrition-related anemia in HIV and/or TB infected/coinfected adults [5, 6].

Both baseline ambulatory and bedridden functional status of adult HIV patients were statistically positively associated with the odds of developing TB/HIV-associated anemia. Though literature on this variable was scarce, it is clear that patients with advanced functional status (i.e., ambulatory and bedridden) might tend to be affected by different opportunistic infections that could negatively affect iron concentration and absorption in which iron deficiency anemia could account for the highest share of anemia in the current study.

Similarly, HIV adults of ART naïve with WHO clinical stage III and IV were more likely to develop TB/HIV-associated anemia than those with clinical stage I/II. The result of the current study was supported by the findings of other studies conducted in Uganda and South Africa which have been reported that the odds of developing anemia was increasing as patients clinical stage advanced from stage I/II to III and then IV [32, 33]. The key reasons could be as HIV infection was advanced from the early stages (I or II) to III/IV–there would be marked weight loss (>10% of total weight), consistent diarrhea, opportunistic infections and then advancement to AIDS defining illness, and other complications [38]. Thus, all these advanced infections and complications during the advanced clinical stages (III and IV) might lead to severe anemia in particular and anemia in general following excessive fluid loss due to prolonged diarrhea in stage-III and occurrence of different opportunistic infections in stage-IV mostly that might suppress the immune system ultimately.

Despite an appropriate study designed, the current study was not free of limitations. The study population were considered as TB/HIV coinfected adults at baseline regardless of the exact time of TB development. The pregnancy status of women at baseline was not known, and thus, the anemia classification did not consider pregnancy status for women that could underestimate the prevalence of anemia in female participants. Most literature used in the current study was HIV-associated anemia irrespective of TB status at enrollment since studies assessed TB/HIV-associated anemia were scarce.

## 5. Conclusions

In the current study, approximately six in ten TB/HIV coinfected adults had laboratory evidence of anemia with almost one-ninth, and half of the anemia cases were severe and moderate anemia, respectively, at baseline. Low baseline BMI, ambulatory functional status, bedridden functional status, WHO clinical stages III and IV increased the odd of developing TB/HIV-associated anemia. Thus, curious attention has to be given for severe anemia in particular, and it has to be managed accordingly so as to limit anemia/severe anemia associated bad outcomes of TB/HIV coinfection most importantly death.

## Figures and Tables

**Table 1:** Baseline socio-demographic characteristics of TB/HIV coinfected patients initiated ART in two public hospitals of Mekelle, Ethiopia, 2009–2016 (*n* = 305).

Socio-demographic characteristic	Severity of anemia				Prevalence rate (number of anemia cases per 100 TB/HIV coinfected adults)	*P* value
	Nonanemia *n* (%)	Mild anemia *n* (%)	Moderate anemia *n* (%)	Severe anemia *n* (%)		
*Sex*						
Male	47 (31.8)	57 (38.5)	37 (25.0)	7 (4.7)	68.2	0.001
Female	78 (49.7)	18 (11.5)	49 (31.2)	12 (7.6)	50.2	

Baseline age in years (median & IQR)					35 (29–40)	0.320

*Educational status*						
No education	25 (38.5)	15 (23.1)	21 (32.3)	4 (6.2)	61.5	0.194
Primary	30 (33.3)	24 (26.7)	31 (34.4)	5 (5.6)	66.7	
Secondary	57 (47.9)	24 (20.2)	28 (23.5)	10 (8.4)	52.1	
Tertiary	13 (41.9)	12 (38.7)	6 (19.4)	0 (0.0)	58.1	

*Marital status*						
Single	30 (43.5)	14 (20.3)	21 (30.4)	4 (5.8)	56.5	0.639
Married	55 (42.6)	34 (26.4)	30 (23.3)	10 (7.8)	57.4	
Others^a^	40 (37.4)	27 (25.2)	35 (32.7)	5 (4.7)	62.6	

*Religion*						
Orthodox	122 (41.6)	72 (24.6)	83 (28.3)	16 (5.5)	58.4	0.251
Muslim	3 (25.0)	3 (25.0)	3 (25.0)	3 (25.0)	75.0	

*Place of residence*						
Urban	90 (40.5)	51 (23.0)	69 (31.1)	12 (5.4)	59.5	0.797
Rural	35 (42.2)	24 (28.9)	17 (20.5)	7 (8.4)	57.8	

Others^a^: widowed/divorced/separated, IQR: interquartile range.

**Table 2:** Baseline clinical characteristics of TB/HIV coinfected patients initiated ART in two public hospitals of Mekelle, Ethiopia, 2009–2016 (*n* = 305).

Baseline clinical characteristics	Severity of anemia				Prevalence rate (number of anemia cases per 100 TB/HIV coinfected adults)	*P* value
	Nonanemia *n* (%)	Mild anemia *n* (%)	Moderate anemia *n* (%)	Severe anemia *n* (%)		
*Functional status*						
Working	76 (53.9)	33 (23.4)	28 (19.9)	4 (2.8)	46.1	<0.001
Ambulatory	34 (30.9)	30 (27.3)	39 (35.50)	7 (6.4)	69.1	
Bedridden	15 (27.8)	12 (22.2)	19 (35.2)	8 (14.8)	72.2	

BMI in kg/m^2^ (median and IQR)					17.4 (15.6–19.3)	<0.001

*WHO clinical stage*						
I/II	23 (71.9)	6 (18.7)	3 (9.4)	0 (0.0)	28.1	<0.001
III	56 (42.7)	31 (23.7)	38 (29.0)	6 (4.6)	57.3	
IV	46 (32.4)	38 (26.8)	45 (31.7)	13 (9.2)	67.6	

*CD4+ cell count (in cells/mm* ^*3*^)						
≤200	90 (36.0)	64 (25.6)	79 (31.6)	17 (6.8)	64.0	<0.001
>200	35 (63.6)	11 (20.0)	7 (12.73)	2 (3.64)	36.4	

Serum ALT in IU/l (median and IQR)					34.5 (24–51)	0.871

Serum AST in IU/l (median and IQR)					40 (28–60)	0.122

ALT: alanine transferase, AST: aspartate transferase, BMI: body mass index.

**Table 3:** Determinants of TB/HIV-associated anemia of patients initiated ART in two public hospitals of Mekelle, Ethiopia, 2009–2016 (*n* = 305).

Independent variables	Category	AOR (95% CI)	Standard error		*P* value
Sex	Male	1.000 (reference category)			
	Female	0.380 (0.226; 0.640)	0.101		<0.001^*∗∗*^

BMI (kg/m^2^)		0.913 (0.836; 0.998)	0.041		0.044^*∗*^

Functional status	Working	1.000 (reference category)			
	Ambulatory	2.139 (1.189; 3.846)	0.640		0.011^*∗*^
	Bedridden	2.208 (1.002; 4.863)	0.889		0.049^*∗*^

WHO clinical stage	I Or II	1.000 (reference category)			
	III	2.565 (1.030; 6.384)	1.193		0.043^*∗*^
	IV	2.590 (1.006; 6.669)	1.250		0.049^*∗*^

CD4+ cell count (cells/mm^3^)	>200	1.000 (reference category)			
	≤200	1.666 (0.830; 3.328)	0.588		0.148

Serum AST level (IU/l)		1.003 (0.997; 1.009)	0.003		0.369

AOR: adjusted odds ratio, BMI: body mass index, CI: confidence interval, ^*∗*^: significant at 5% level of significance, ^*∗∗*^: significant at 1% level of significance.

## Data Availability

The datasets used to support the findings of this study are available from the corresponding author uponrequest.

## Abbreviations

AOR: Adjusted odds ratio
AIDS: Acquired immunodeficiency syndrome
ALT: Alanine transferase
ART: Antiretroviral therapy
AST: Aspartate transferase
BMI: Body mass index
CI: Confidence interval
HAART: Highly active antiretroviral therapy
HIV: Human immunodeficiency virus
LTFU: Lost to follow-up
SSA: Sub-Saharan Africa
TB: Tuberculosis
WHO: World Health Organization.
